# Unveiling the Role of the Fatty Acid Binding Protein 4 in the Metabolic-Associated Fatty Liver Disease

**DOI:** 10.3390/biomedicines10010197

**Published:** 2022-01-17

**Authors:** Juan Moreno-Vedia, Josefa Girona, Daiana Ibarretxe, Lluís Masana, Ricardo Rodríguez-Calvo

**Affiliations:** 1 Vascular Medicine and Metabolism Unit, Research Unit on Lipids and Atherosclerosis, “Sant Joan” University Hospital, Universitat Rovira i Virgili, Institut de Investigació Sanitaria Pere Virgili (IISPV), 43204 Reus, Spain; juanmorenovedia@gmail.com (J.M.-V.); josefa.girona@urv.cat (J.G.); daianaig@hotmail.com (D.I.); 2 Spanish Biomedical Research Centre in Diabetes and Associated Metabolic Disorders (CIBERDEM), 28029 Madrid, Spain

**Keywords:** MAFLD, MASH, liver steatosis, adipokines, FABP4

## Abstract

Metabolic-associated fatty liver disease (MAFLD), the main cause of chronic liver disease worldwide, is a progressive disease ranging from fatty liver to steatohepatitis (metabolic-associated steatohepatitis; MASH). Nevertheless, it remains underdiagnosed due to the lack of effective non-invasive methods for its diagnosis and staging. Although MAFLD has been found in lean individuals, it is closely associated with obesity-related conditions. Adipose tissue is the main source of liver triglycerides and adipocytes act as endocrine organs releasing a large number of adipokines and pro-inflammatory mediators involved in MAFLD progression into bloodstream. Among the adipocyte-derived molecules, fatty acid binding protein 4 (FABP4) has been recently associated with fatty liver and additional features of advanced stages of MAFLD. Additionally, emerging data from preclinical studies propose FABP4 as a causal actor involved in the disease progression, rather than a mere biomarker for the disease. Therefore, the FABP4 regulation could be considered as a potential therapeutic strategy to MAFLD. Here, we review the current knowledge of FABP4 in MAFLD, as well as its potential role as a therapeutic target for this disease.

## 1. Background

Non-alcoholic fatty liver disease (NAFLD) represents the leading cause of chronic liver disease worldwide, reaching epidemic proportions [1]. Indeed, the global prevalence of NAFLD affects currently ~25% of the general population around of world [2], which is even higher in morbidly obese individuals [3] and in type 2 diabetic patients [4,5]. Additionally, NAFLD has been proposed as an important risk factor for obesity-related conditions and cardiovascular disease [6,7,8].

At a clinical level, NAFLD can be defined as a progressive disease encompassing from the so-called non-alcoholic fatty liver (NAFL) to the non-alcoholic steatohepatitis (NASH). Whereas NAFL is defined as a disproportionate accumulation of triglycerides as lipid droplets in more than 5% of hepatocytes in the absence of excessive alcohol consumption (<20 g/day in females and <40 g/day in males), NASH is a more advanced stage characterized by lobular inflammation, hepatocyte ballooning and cell death, and increased risk for liver fibrosis [9]. NASH can irreversibly progress to end-stage liver disease, leading to liver transplantation [10,11,12,13], including cirrhosis and hepatocellular carcinoma [14]. In order to highlight the strong pathophysiological relationship between steatosis and metabolic dysfunction, a change of nomenclature from NAFLD to metabolic-associated fatty liver disease (MAFLD) has recently been proposed [15]. Accordingly, NASH has been renamed as metabolic-associated steatohepatitis (MASH).

Although MAFLD is recognized as the main contributor for chronic liver disease, it remains underdiagnosed and undertreated because of the lack of effective methods for its diagnosis and monitoring. MAFL can be diagnosed by imaging techniques, including ultrasound, computed tomography, or magnetic resonance imaging (for review, see [16]). Nevertheless, these imaging techniques are unreliable. MASH may be suspected when aminotransferases are elevated in patients with liver steatosis, although serum ALT in MASH patients has a poor sensitivity (for review see [16]). As MASH can progress to fibrosis, MAFLD fibrosis score FIB-4 and elastography are widely used to identify advanced fibrosis. Nevertheless, progression to advanced stages of MAFLD is often unnoticed as most patients are asymptomatic. Therefore, MASH diagnosis is often made serendipitously, and high-invasive liver biopsy is required for a definitive diagnosis [16]. Unfortunately, this approach has several associated limitations, including invasiveness, potentially life-threatening complications, or sampling variability, among others, which makes it unsuitable for monitoring the disease. Despite the increasing knowledge of the molecular mechanisms underlying both the onset and progression of MAFLD, once diagnosed, the therapeutic options for this disease are scarce, as no specific drugs for its treatment have been approved yet. Therefore, the search for potential biomarkers allowing for the diagnosis and monitoring of the disease, as well as new molecular therapeutic targets, has been a very active field of research in recent years.

Because the steady increase in MAFLD prevalence has grown in parallel with the economic burden [17], new approaches for its diagnosis and clinical management are of the utmost importance, not only from a health, but also from a socio-economic point of view. Given the close relationship between MAFLD and obesity-related conditions [6], several adipokines could act as relevant players in the adipose tissue/liver cross-talk. Among these adipokines, fatty acid binding protein 4 (FABP4) has been recently related to ectopic fat deposition in several tissues, including the liver [18,19,20,21,22,23,24,25,26,27,28,29,30]. Here, we will review the role of FABP4 as a potential biomarker, contributor, and therapeutic target for the MAFLD.

## 2. Etiopathogenesis of MAFLD: Role of White Adipose Tissue

Despite MAFLD having been identified in a sizable percentage of individuals with a body mass index (BMI) < 23 [31], epidemiologic studies have shown that it is closely associated with obesity and other metabolic comorbidities, including dyslipidemia and diabetes [32,33,34]. Specifically, ectopic fat accumulation in the liver predisposes to liver injury and MAFLD progression [35,36]. An increased liver triglyceride content may be due to an enhanced non-esterified fatty acid uptake as a result of an increased lipolysis in adipose tissue mediated by insulin resistance in patients with obesity, metabolic syndrome, or type 2 diabetes, among other conditions [37,38]. Additionally, hyperinsulinemia induces liver triglyceride synthesis through activation of de novo lipogenesis. Specifically, insulin activates the sterol regulatory element binding protein-1c (SREBP-1c), a key transcription factor involved in de novo lipogenesis regulation, by promoting the SREBP-SREBP-1c cleavage activating protein (SCAP) complex dissociation from the insulin-induced gene (Insig), and the subsequent SREBP-1c cleavage by site 1 (S1P) and site 2 (S2P) proteases in the Golgi apparatus [39]. de novo lipogenesis is also activated as a consequence of overnutrition based on a high intake of fats and simple sugars. Both mechanisms (increased liver fatty acid uptake and de novo lipogenesis) lead to liver triglyceride deposition, exceeding the mechanisms for fat removal, such as very low-density lipoprotein (VLDL) secretion and mitochondrial fatty acid β-oxidation [37,38], further promoting liver triglyceride [40,41] and other lipid molecule accumulation. Among these lipid molecules, other than triglycerides, it is possible to find diacylglycerols [42], ceramides [43,44], lysophosphatidyl cholines [45,46], and cholesterol [47]. The molecular mechanisms by which the ectopic accumulation of triglycerides and additional bioactive lipid intermediates in the liver lead to MAFLD-to-MASH progression are multiple, and are not completely understood. The initial “two-hit” hypothesis, suggesting that MASH results from a second insult in triglyceride-sensitized livers, has been substituted by the current “multiple-hit” hypothesis, considering different insults acting synergistically to induce progression to MASH. Among the lipid-mediated cellular processes leading to the disease progression is the activation of several intracellular signal cascades mediated by Ser/The-kinases involved in the regulation of cellular proliferation [48], mitochondrial dysfunction [49], inflammation [49,50], fibrogenesis [50], or endoplasmic reticulum stress-induced apoptosis [51], among others.

It is estimated that white adipose tissue is a source of roughly 60% of the triglycerides found in the liver of MAFLD patients [52,53], thereby highlighting the role of this tissue as a relevant player in MAFLD pathogenesis. Actually, it has been shown that MAFLD prevalence correlates with the obesity rate and increases with the body mass index [54,55]. Besides providing non-esterified fatty acids for liver triglyceride synthesis, adipocytes act as endocrine organs releasing a wide range of biologically active molecules termed adipokines [56], to the bloodstream, which take part in the cross-talk between adipose tissue and peripheral organs [57]. Among the most studied adipokines released from white adipose tissue are adiponectin, leptin, resistin, plasminogen activator inhibitor-1 (PAI-1), and tumor necrosis factor α (TNFα). Additionally, FABP4 has been proposed as a potential biomarker/causal agent for ectopic fat deposition in non-adipose tissues [58], including the liver [18,20,21,22,23,24,25,30,59,60,61,62,63].

## 3. Fatty Acid Binding Protein 4

FABP4, also termed adipocyte FABP (A-FABP) or adipocyte P2 (aP2), is an intracellular lipid-binding protein mainly expressed in adipocytes [64], but also in macrophages [65], and dendritic [66] and endothelial cells [67] to a lesser extent. Additionally, the *FABP4* gene expression has been found to be upregulated in the livers of morbidly obese patients in the context of insulin resistance [68] and in patients with different MAFLD severities [59,60,61,62,63].

FABP4 belongs to a family of small (~15 kDa) intracellular lipid chaperones highly expressed in active lipid metabolic tissues, including specific forms for liver (FABP1), intestines (FABP2), heart (FABP3), epidermis (FABP5), ileum (FABP6), brain (FABP7), myelin (FABP8), and testis (FABP9). Although the different members of the FABP family show variable sequence homologies (15–70%) [69], all of them share a similar protein structure characterized by two orthogonal five-stranded β-sheets and a 10-stranded anti-parallel β-barrel [69,70,71], which form an interior water-filled binding cavity [72] that allows for the transport of long-chain non-esterified fatty acids [73]. Given its similar ligand-binding properties, the FABP members reversibly bind with nanomolar affinity to hydrophobic ligands, including non-esterified saturated and unsaturated long-chain fatty acids, and additional ligands such as eicosanoids, lysophospholipids, prostaglandins, or bile acids [73,74]. In this way, FABPs are actively involved in the transport of fatty acids to specific cellular organelles, including the mitochondria, peroxisomes, nucleus, and endoplasmic reticulum, thereby taking part in the regulation of lipid-mediated cellular responses [71,75,76,77,78]. Among them, FABP4 takes part in the control of transcriptional regulation, cellular signaling, lipid droplets storage, lipid oxidation, synthesis of membranes, regulation of enzymatic activity [71], conversion of fatty acids to eicosanoids, and leukotrienes stabilization [79,80].

Human FABP4 is a polypeptide of 132 amino acids with a molecular mass of 14.6 kDa encoded by the human *FABP4* gene (GenBank accession number NM_024406). At a transcriptional level, the *FABP4* expression is controlled by the CCAAT/enhancer-binding protein (CEBP) [81] and peroxisome proliferator-activated receptor γ (PPARγ) [82,83]. The *FABP4* expression is further controlled by the cyclic adenosine monophosphate (cAMP) through a mechanism involving the relief of a negative regulatory element in the *FABP4* promoter [84]. FABP4 was proposed as an adipocyte differentiation marker [84,85,86], as it is enhanced during adipocyte differentiation [87]. Additionally, the *FABP4* expression is strongly induced by a wide range of proinflammatory stimuli during differentiation from monocytes to macrophages [65,88,89,90,91,92]. Specifically, in macrophages, FABP4 takes part in cholesterol ester storage, foam cell formation, and inflammatory responses, through the regulation of the I kappa B kinase-nuclear factor kappa B (IKK-NF-κB) and Jun N-terminal kinase 1- activator protein-1 (JNK-AP-1) pathways [93,94]. On the other hand, FABP4 is able to enhance the hydrolytic activity of hormone-sensitive lipase (HSL) [95,96] and it regulates the PPARγ transcriptional activity transporting specific PPARγ agonists to the nucleus [97,98]. Curiously, only PPARγ, and no other FABP4 ligands, lead to FABP4 nuclear localization by exposing the FABP4 nuclear localization signal in the three-dimensional structure of the protein [98,99].

Therefore, FABP4 regulates both lipid metabolism and insulin sensitivity [100]. Although FABP4 lacks an N-terminal secretory signal sequence [71], it can be released from adipocytes in association with lipolysis through additional mechanisms [101,102,103,104]. During lipolysis, the HSL activation by both β-adrenergic receptor-mediated adenylyl cyclase (AC)–protein kinase A (PKA) and natriuretic peptide receptor-A (NPR-A)-mediated guanylyl cyclase (GC)–protein kinase G (PKG) induces FABP4 secretion from adipocytes [104]. Nevertheless, the FABP4 circulating levels are declined postprandially by a mechanism involving FABP4 secretion by insulin-induced anti-lipolytic signaling [104]. Therefore, it seems that under physiological conditions, FABP4 would be involved in the adipocyte extracellular mobilization of fatty acids. Nevertheless, the enhanced lipolysis associated with insulin resistance may contribute to increased FABP4 secretion from adipocytes, which in turn would contribute to further activation of HSL, promoting a vicious cycle that would lead to increase levels of circulating FABP4. Although a FABP4 threshold level should be defined for each pathological condition, it is worth noting that it is strongly associated with adiposity [105,106] and with a wide range of metabolic diseases, including endothelial dysfunction [107], metabolic syndrome [83,108], and type 2 diabetes [109], as well as cardiovascular diseases (for review see [110]). Furthermore, exogenous FABP4 is able to interact with the cell membrane of different cell types [77] through a mechanism involving the physical interaction with cytokeratin 1 [77,78]. Therefore, circulating FABP4 directly takes part in the cross-talk between adipose tissue and peripheral tissues, acting as an adipokine in several organs, including the liver.

## 4. FABP4 as Potential Biomarker for MAFLD

**Figure 1:**
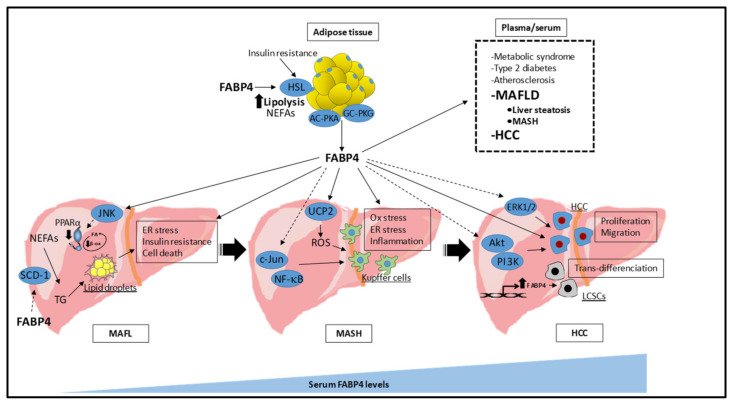
Potential mechanisms of FABP4 involvement in MAFLD. FABP4 increases adipose tissue lipolysis through HSL activation, thereby releasing NEFAs to the bloodstream that are taken by the liver to synthesize triglycerides. Additionally, FABP4 is also released to the bloodstream through a non-conventional mechanism and its circulating levels are associated with a large number of metabolic diseases, including MAFLD and hepatocellular carcinoma. In the liver, FABP4 contributes to triglyceride synthesis and its accumulation as lipid droplets, potentially by regulating SCD-1. FABP4 contributes to liver endoplasmic reticulum stress, insulin resistance, and cellular death. Additionally, FABP4 may contribute to the oxidative stress induction of endoplasmic reticulum stress and inflammation in Kupffer cells, at least partially activating JNK and NF-κB and the UCP2-induced ROS production, altogether contributing to MASH progression, and increases proliferation and migration of hepatocellular carcinoma cells, potentially through activation of Akt/PI3K and ERK1/2. The liver *FABP4* mRNA levels have been found to be increased in hepatocellular carcinoma with liver steatosis, and contribute to liver cancer stem cells trans-differentiation. AC-PKA, adenylyl cyclase–protein kinase A; Akt, protein kinase B; β-ox, beta oxidation; ER, endoplasmic reticulum; ERK1/2, extracellular regulated kinase 1/2; FA, fatty acids; FABP4, fatty acid binding protein 4; GC-PKG, guanylyl cyclase–protein kinase G; HCC, hepatocellular carcinoma; HSL, hormone-sensitive lipase; JNK, Jun N-terminal kinase 1; LCSCs, liver cancer stem cells; TG, triglycerides; MAFL, metabolic-associated fatty liver; MAFLD, metabolic-associated fatty liver disease; MASH, metabolic-associated steatohepatitis; NEFAs, non-esterified fatty acids; NF-κB, nuclear factor kappa B; Ox, oxidative; PI3K, phosphatidylinositol 3 kinase; ROS, reactive oxygen species; SCD-1, stearoyl-CoA desaturase-1; UCP2, uncoupling protein 2.

The role of FABP4 in metabolic and cardiovascular diseases has been extensively explored [83,108,109,110]. Nevertheless, studies reporting its role in MAFLD have yield somewhat conflicting results. Whereas the serum FABP4 levels were found to be inversely associated with liver steatosis in a study performed in an elderly population (> 65 years) [111], others have shown increased serum FABP4 levels in non-elderly MAFLD patients [20,21,22,23,24,25,30,112,113]. These apparently contradictory results may be explained because in the elderly population, the regulation of energy homeostasis showed some differences compared to young populations, partly due to the chronic inflammatory status induced by changes in metabolism related to specific body composition modifications occurring with age (for a review, see [114]). Studies performed in non-elderly individuals showed that patients with higher FABP4 levels are more likely to have MAFLD compared with patients with lower FABP4 levels [21,24]. Actually, serum FABP4 levels increased in a stepwise fashion with disease severity [113]. Additionally, *FABP4* gene expression was found to be upregulated in the liver biopsies from patients with extreme steatosis without histological signs of inflammation, compared with subjects with a low liver fat content [60]. Serum FABP4 levels were positively associated with suspected liver steatosis assessed by the fatty liver index (FLI) in both normal glucose tolerance subjects [23] and patients at increased cardiometabolic risk, including diabetic, obese, and metabolic syndrome patients [30]. The independent association between FABP4 and MAFLD had been previously reported in both apparently healthy individuals [21] and in diabetic patients [20]. Furthermore, baseline serum FABP4 levels were associated with FLI in healthy individuals after 4 years of follow-up, thereby proposing FABP4 as a predictive indicator for MAFLD [22]. The relationship between disease progression and elevated serum FABP4 levels was further reported in patients with histologically confirmed MAFLD [25]. Additionally, plasma FABP4, but no other adipokines, was associated with a specific liver triglyceride pattern, mainly composed by long-chain saturated fatty acids, in a murine model of MAFLD induced by a high-fat diet [18], thus suggesting that FABP4 may influence not only the fat content, but also the specific lipid composition related to the onset and progression of the disease. On the other hand, serum FABP4 has been associated with hallmarks of MASH, including gamma-glutamyl transpeptidase (GGT) and ultra-sensitive C reactive protein (usCRP) [30], and it has been proposed as a predictive factor for intrahepatic inflammation and fibrosis confirmed by liver histology [25]. Moreover, the serum FABP4 levels have been positively correlated to other pro-inflammatory cytokines, such as TNFα, in apparently healthy subjects with MAFLD assessed by abdominal ultrasonography [21], thereby supporting a role of FABP4 not only as a fatty liver biomarker, but also reflecting inflammatory states related to advanced stages of the disease. Actually, the *FABP4* expression was higher in visceral adipose tissue from MASH than non-MASH patients [115]. Additionally, *FABP4* mRNA levels were found to be upregulated in the livers from MASH patients [61,62,63], and it has been proposed as an efficient classifier of mild-MAFL and MASH [63]. Moreover, a high liver expression of *FABP4* has been associated with increased risk for progression to MASH [59]. On the other hand, the serum FABP4 levels have been found increased in patients with hepatocellular carcinoma, thereby suggesting a pivotal role as a potential biomarker not only for MAFLD, but also for end-stage liver disease [112]. Therefore, FABP4 may not only reflect liver steatosis, but also advanced stages of MAFLD (Figure 1).

## 5. FABP4 as Active Player in the MAFLD

Despite the huge evidence reporting a close relationship between FABP4 and MAFLD, the interventional studies propose FABP4 as a biomarker, rather than as an etiological factor directly involved in the disease progression. However, increasing experimental studies suggest a causal role of FABP4 in disease development. Nevertheless, the molecular mechanisms by which FABP4 contributes to MAFLD are beginning to emerge and are not completely understood yet. As mentioned above, roughly ~60% of the liver triglycerides in MAFLD come from adipocytes [52,53]. Specifically, FABP4 may regulate lipolysis in white adipose tissue [101,102,103] by activating HSL [96]. Therefore, FABP4 may contribute to liver triglycerides synthesis by regulating the non-esterified fatty acids’ release from adipocytes. Furthermore, exogenous FABP4 is internalized by interacting with the cell membrane of different cell types through a mechanism involving binding with cytokeratin 1 [77,78], and regulating several cellular responses [19,58,76,78,116]. The effect of exogenous FABP4 activating some of these cellular pathways was attenuated in the presence of a siRNA against cytokeratin 1 [78], thereby highlighting the physical interaction between exogenous FABP4 and cytokeratin 1 as a relevant mechanism driving the exogenous FABP4 uptake and responses at a cellular level. Focusing in the liver, exogenous FABP4 increased the intracellular lipid content in a liver cell line, thereby showing a direct role of FABP4 in liver cells [19]. Accordingly, the combined deficiency of both *FABP4* and *FABP5* was protected in front of the fatty liver [26,27], at least partially by suppressing the liver stearoyl-CoA desaturase-1 [27]. The lipotoxicity induced by FABP4 in the liver cells was related to an activation of endoplasmic reticulum stress, an impairment of the insulin signaling pathway, and increased cellular mortality [19]. The FABP4-induced endoplasmic reticulum stress-associated intracellular lipid content in liver cells was in line with the activation of JNK [19]. As JNK takes part in the regulation of the mitochondrial fatty acid β-oxidation [117] by downregulating peroxisome proliferator-activated receptor α (PPARα) [118], FABP4 may contribute to intracellular lipid accumulation in liver cells through the regulation of these pathways. Endoplasmic reticulum stress activation by FABP4 has been further reported in macrophages [119], proposing endoplasmic reticulum stress as a key mechanism through which FABP4 takes part in the progression to MASH. Specifically, the molecular mechanism behind the FABP4 regulation of endoplasmic reticulum stress and/or inflammation in macrophages involves the uncoupling protein 2 (UCP2)-mediated reactive oxygen species (ROS) regulation [120,121,122]. As ROS is the main culprit of oxidative stress [123], FABP4 may contribute to MASH progression through ROS-induced mitochondrial dysfunction and increasing hepatocellular oxidative damage. It has been proposed that FABP4 is able to integrate both metabolic and immune responses to link inflammatory and lipid-mediated pathways [124]. *FABP4* was found to be one of the top 10 up-regulated genes in a transcriptome signature study performed in MASH patients [62], suggesting a relevant role not only for liver fat deposition, but also for MASH progression. The liver expression of *FABP4* was reduced in the absence of cathepsin B, a cysteine protease found to be upregulated in the livers of MASH mice, supporting its role as an active player in the disease [125]. Specifically in the liver, FABP4 potentiated inflammation in Kupffer cells induced by a high fat high cholesterol diet and contributed to lipopolysaccharide-induced acute liver injury [28], potentially through the activation of NF-κB and JNK [29,93]. The role of FABP4 in liver injury and inflammation was further clarified by using adenovirus-mediated liver *FABP4* overexpression [28]. Additionally, the *FABP4* expression was increased in hepatocellular carcinoma with liver steatosis, thus suggesting an active role of this molecule in the progression to end-stage liver disease [126]. Actually, in vitro studies showed that exogenous FABP4 significantly increased the proliferation and migration of human hepatocellular carcinoma cells [112]. Although the mechanisms responsible for exogenous FABP4 eliciting proliferation and migration of hepatocellular carcinoma cells have not been specifically explored, studies performed in breast cancer cells [127] and prostate cancer cells [128] suggest that exogenous FABP4 may regulate these processes through a mechanism involving membrane binding and internalization, and through the activation of cellular signaling pathways such as Akt/phosphatidylinositol 3 kinase (PI3K) and extracellular regulated kinase 1/2 (ERK1/2), among others. Moreover, a recent study from Yang et al. proposed the activation of the lipoprotein lipase (LPL)/FABP4/carnitine palmitoyl transferase 1 (CPT1) axis as a direct contributor to liver cancer stem cell trans-differentiation during MASH to hepatocellular carcinoma progression [129]. Given that the liver expression of these three genes was up-regulated in the progression of MAFLD/MASH to hepatocellular carcinoma, and its inhibition delayed tumor growth and reduced the sphere-forming, proliferation, and clonality of liver cancer stem cells, it was proposed that the LPL/FABP4/CPT1 axis may regulate tumorigenesis by regulating fatty acid production, transport, and oxidation. Therefore, the LPL/FABP4/CPT1 axis would generate a tumor microenvironment providing fatty acids for building blocks and energy production (i.e., fatty acid oxidization), which is essential for the proliferation of tumor-initiating cells. Altogether, although further studies are warranted in order to fully clarify the role of FABP4 in the cellular responses underlying these processes, increasing evidence highlights an active role of both exogenous and liver-expressed *FABP4* in the onset and progression of the MAFLD and hepatocellular carcinoma (Figure 1).

## 6. FABP4 as a Potential Therapeutic Target for MAFLD

Both clinical and experimental studies have identified FABP4 as a pivotal player, not only as an emerging tool for diagnosis, but also as a relevant actor involved in MAFLD progression. Therefore, reducing the circulating levels of this adipokine may directly impact the onset and progression of the disease. Given that the main source of FABP4 is adipose tissue [71,110], reducing its adipocyte secretion by regulating both AC-PKA and GC-PKG pathways may reduce the impact of the adipose-derived FABP4 on the liver. Additionally, future strategies aimed at blocking the interaction between FABP4 and cytokeratin 1 could decrease the impact of FABP4 on the liver. On the other hand, increasing studies suggest that reducing the liver *FABP4* expression may directly contribute to reducing the impact of MAFLD. Dietary sphingomyelin attenuated hepatic steatosis potentially by downregulating the liver expression of several PPARγ-related genes, including *FABP4* [130]. The combination of n-3 long chain polyunsaturated fatty acids and flavan-3-ols prevented the high-fat high-fructose diet-induced MAFLD and liver *FABP4* expression [131], suggesting an impact of FABP4 in the disease. Moreover, the reduction in the liver expression of *FABP4* by the ubiquitous miR-100 overexpression was in line with the effect of this molecule protecting from high fat diet-induced liver steatosis [132]. The Huang-Qi San, a combination of three traditional Chinese medicines used for the treatment of several diseases, given its anti-oxidant, anti-diabetic, hepatoprotective, immunological and anti-inflammatory properties, reduced both plasma FABP4 levels and liver lipid accumulation in high fat diet-fed rats [133]. Additionally, the Hugan Qingzhi tablet attenuated the high fat diet-induced liver *FABP4* expression [134], suggesting that FABP4 reduction may be involved in its therapeutic effects. Theobromine, a methylxanthine present in cocoa with anti-oxidant, anti-inflammatory, and anti-microbial activities, mitigated liver steatosis and reduced the liver *FABP4* expression in obese mice [135]. Tetrahydrocurcumin, a naturally occurring curcuminoid and a metabolite of curcumin showing properties as an anti-oxidant, anti-inflammatory, anti-cancer, anti-diabetic, and neuroprotective agent, reduced oleic acid-induced lipid accumulation in human hepatocellular carcinoma cells through several mechanisms, including the attenuation of lipogenesis by reducing the protein levels of FABP4, among other lipogenic proteins [136]. As the *FABP4* expression is under the transcriptional control of PPARγ [82,83], the reduction of this transcription factor by Tetrahydrocurcumin [136] may be a molecular mechanism underlying FABP4 reduction by this metabolite. Korean red ginseng, other traditional herbal medicine used to prevent several geriatric diseases due to its therapeutic effects on metabolic disorder (i.e., type 2 diabetes and fatty liver disease), improved MASH-related inflammation by reducing both *FABP4* mRNA and protein levels [137]. Saikosaponin-d, an active extract from *Bupleurum falcatum* used for the treatment of respiratory, inflammatory, and infectious diseases, reduced the liver *FABP4* expression and protected against liver inflammation, endoplasmic reticulum, and oxidative stress [138]. Dieckol, a component of *Ecklonia cava* extracts with beneficial effects in inflammation, oxidation, and adipogenesis, attenuated the high fat diet liver induction of lipid accumulation and reduced the nucleotide-binding oligomerization domain-like receptor family, pyrin domain containing 3 (NLRP3) inflammasome [139]. The mechanisms driving the liver lipid reduction by Dieckol include downregulation of the lipogenic genes, such as *PPARγ* and its target *FABP4*, among others [139]. The reduction in *FABP4* expression, lipid accumulation, and inflammation by fatty acids was also observed in both HepG2 and Drosophila melanogaster models of liver steatosis treated with hydroethanolic extract of *Lampaya medicinalis Phil*. (Verbenaceae) [140], a folk medicine of Chile used to counteract inflammatory diseases. Treatment with recombinant bone morphogenetic protein 9 (BMP9) alleviated hepatic steatosis and decreased liver macrophage infiltration in mice fed with a high fat diet, partially via decreasing the *FABP4* promoter chromatin accessibility and the subsequent reduction in the *FABP4* expression [141]. A therapeutic approach based on increasing the bioavailability of silybin, the major active compound of the antioxidant serum Silymarin used in the prevention and treatment of liver injury, improved MASH-associated oxidative stress, inflammation, and lipid accumulation, concomitantly to the reduction in the liver expression of both *PPARγ* and its target *FABP4* [142]. Additionally, the dual peroxisome proliferator-activated receptor α/δ (PPAR α/δ) agonist GFT505 (elafibranor), which has shown a potential therapeutic effect against NASH in clinical trials, reduced the liver *FABP4* expression and ameliorated hepatic steatosis, inflammation, and fibrosis in a choline-deficient, L-amino acid-defined high-fat diet-induced MASH model [143]. Furthermore, the studies performed in *FABP4*-deficient mice have proposed the FABP4 inhibition as a potential therapeutic approach against this disease [27]. Among the synthetic FABP4 inhibitors developed to date [71,144,145,146,147,148,149,150,151,152], BMS309403, which competitively inhibits the binding of endogenous fatty acids [29,71,146], protects against several metabolic disturbances [29,153], including fatty liver disease [28,29]. Specifically, BMS309403 alleviated both lipopolysaccharide-induced acute liver injury and high fat high cholesterol diet-induced MASH, at least partially, by decreasing the activation of both c-Jun and NF-κB [28]. Accordingly, this compound attenuated the fatty acid infiltration and the liver triglyceride content in ob/ob mice [29]. Moreover, FABP4 inhibition by HTS01037 attenuated the pro-inflammatory profile in macrophages [120], supporting the use of FABP4 inhibitors not only for the fatty liver, but also for the treatment of MASH. BMS309403 further reduced liver carcinogenesis in Stelic Animal Model (STAM) mice and reduced the sphere-forming, proliferation, and clonality of liver cancer stem cells, proposing FABP4 inhibition as an emerging therapeutic approach for end-stage liver disease [129].

**Figure 2:**
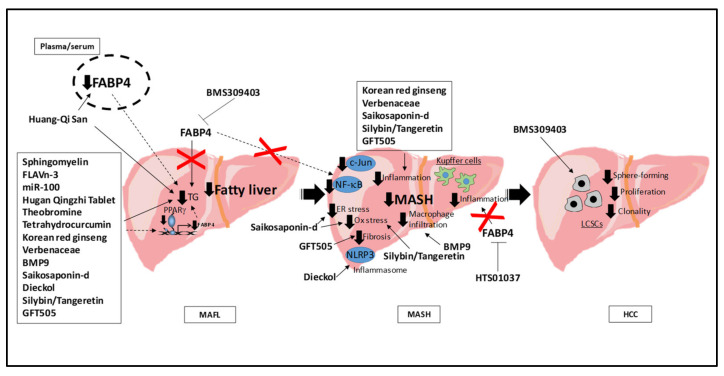
Pharmacological approaches targeting FABP4 for MAFLD. Several therapeutic approaches potentially reduce MAFLD by reducing *FABP4* levels via *PPARγ* down-regulation. Dietary sphingomyelin, FLAVn-3, miR100, Theobromine, Tetrahydrocurcumin, and Hugan Qingzhi tablets reduce both liver steatosis and liver *FABP4* expression. Huang-Qi San also reduces plasma FABP4 levels. The liver *FABP4* mRNA levels are additionally reduced by other compounds improving MASH-related features. Liver inflammation is attenuated by Korean red ginseng, Verbenaceae, Saikosaponin-d, the combination of Silybin/Tangerin, and GFT505. Additionally, Dieckol reduces NLRP3 inflammasome and BMP9 attenuates macrophage infiltration. Oxidative stress is reduced by Saikosaponin-d and the combination of Silybin/Tangerin, endoplasmic reticulum stress by Saikosaponin-d and fibrosis by GFT505. The selective FABP4 inhibitor BMS309403 reduces fatty liver and inflammation through the inhibition of JNK and NF-κB, and reduced liver carcinogenesis by reducing thesphere-forming, proliferation, and clonality of liver cancer stem cells, potentially through Akt and ERK1/2 activation. HTS01037, another FABP4 inhibitor, reduces inflammation in Kupffer cells. Therefore, approaches aimed at FABP4 inhibition/reduction may be considered as promising therapeutic tools against MAFLD. BMP9, bone morphogenetic protein 9; ER, endoplasmic reticulum; HCC, hepatocellular carcinoma; FABP4, fatty acid binding protein 4; FLAVn-3, flavan-3-ols long-chain n-3 poly-unsaturated fatty acids (LC-PUFA); JNK, Jun N-terminal kinase 1; LCSCs, liver cancer stem cells; TG, triglycerides; MAFL, metabolic-associated fatty liver; MAFLD, metabolic-associated fatty liver disease; MASH, metabolic-associated steatohepatitis; NF-κB, nuclear factor kappa B; NLRP3, nucleotide-binding oligomerization domain-like receptor family, pyrin domain containing; Ox, oxidative; PPARγ, peroxisome proliferator-activated receptor γ.

Therefore, approaches targeting FABP4 inhibition/reduction, including synthetic FABP4 inhibitors [71,144,145,146,147,148,149,150,151,152], natural FABP4 inhibitors [154], FABP4 neutralizing antibodies [155,156], or short-hairpin RNAs (shRNAs) targeting *FABP4* expression [157], may be considered as promising therapeutic tools against MAFLD (Figure 2). Nevertheless, further studies aimed to determine the efficacy and safety of FABP4 inhibitors are needed before considering them as a realistic option for the treatment of MAFLD.

## 7. Concluding Remarks

MAFLD is a progressive chronic disease originated as a consequence of a disproportionate accumulation of the liver triglyceride content, which remains underdiagnosed, as effective non-invasive methods for its diagnosis and staging are lacking. Once diagnosed, therapeutic options are scarce as no specific drugs for the different stages of MAFLD have been approved yet. Adipose tissue appears as a key tissue involved in MAFLD onset and progression, as it is the main source of non-esterified fatty acids for the liver de novo triglyceride synthesis, as well as of a wide range of adipokines and inflammatory mediators involved in progression to MASH. Among the adipocyte-derived molecules, increasing evidence has proposed FABP4 not only as a potential biomarker reflecting fatty liver and additional features of advance stages of MAFLD, but also as a causal agent involved in disease progression and as a potential molecular target for the treatment of this disease. Increasing evidence suggests that FABP4 reduction/inhibition may be considered as a promising therapeutic approach for both MAFLD and end-stage liver disease such as hepatocellular carcinoma. Nevertheless, further studies are needed to fully explore the role of FABP4 as a biomarker able to discriminate the disease severity, and additional studies are necessary to explore the efficacy and the safety of the FABP4 inhibitors before considering them as a realistic therapeutic option for the treatment of the MAFLD.

## Data Availability

Exclude this statement.
